# Verification of Underlying Genetic Cause in a Cohort of Russian Patients with Familial Hypercholesterolemia Using Targeted Next Generation Sequencing

**DOI:** 10.3390/jcdd7020016

**Published:** 2020-05-14

**Authors:** Anna E. Semenova, Igor V. Sergienko, Diego García-Giustiniani, Lorenzo Monserrat, Anna B. Popova, Diana N. Nozadze, Marat V. Ezhov

**Affiliations:** 1 National Medical Research Center of Cardiology, 121552 Moscow, Russia; igorcardio@mail.ru (I.V.S.); anna.b.popova@gmail.com (A.B.P.); dana7.81@mail.ru (D.N.N.); marat_ezhov@mail.ru (M.V.E.); 2 Health in Code SL, Clinical Department, 15006 A Coruña, Spain; diego.garcia@healthincode.com (D.G.-G.); lorenzo.monserrat@healthincode.com (L.M.)

**Keywords:** familial hypercholesterolemia, next generation sequencing, LDLR, Dutch lipid clinic network criteria, cardiovascular risk

## Abstract

Russian patients with familial hypercholesterolemia (FH) were screened for pathogenic mutations using targeted next generation sequencing. Genetic testing was performed in 52 probands with definite or probable FH based on the Dutch lipid clinic network criteria (DLCN score ≥ 6). Blood samples were studied by massive parallel sequencing (Illumina HiSeq 1500 platform) using a custom capture library related to dyslipidemia and premature atherosclerosis. Mutations considered to be responsible for monogenic FH were identified in 48% of the probands: 24 with mutations in the *LDLR* gene and two with a mutation in the *APOB* gene. There were 22 pathogenic/likely pathogenic mutations in *LDLR*, eight of which have not been previously described in the literature. Four patients with a clinical picture of homozygous FH had two heterozygous *LDLR* mutations. Although mutation-negative patients had highly elevated total cholesterol and low-density lipoprotein cholesterol levels, only half of them had a family history of hypercholesterolemia. With respect to heterozygous FH, mutation-positive patients had higher maximum total cholesterol levels (*p* = 0.01), more severe carotid atherosclerotic lesions, and a higher percentage of premature peripheral artery disease (*p* = 0.03) than mutation-negative ones. However, the number of patients who suffered from myocardial infarction was similar between the two groups.

## 1. Introduction

Familial hypercholesterolemia (FH) is a condition mostly associated with pathogenic mutations in the low-density lipoprotein receptor (*LDLR*), apolipoprotein B (*APOB*) and proprotein convertase subtilisin/kexin type 9 (*PCSK9*) genes. This disease is highly underdiagnosed and undertreated in the Russian Federation and its true prevalence remains unknown [1]. An analysis of the population in West Siberian, Russia, showed an estimated prevalence of about 1:400 for clinically diagnosed definite FH and 1:108 for both definite and probable FH in combination [2], which is consistent with the estimated prevalence of FH in Europe and the USA [3,4,5,6]. The recent development of Russian FH Registry is intended to bring more attention to the problem FH represents in Russia (ClinicalTrials.gov Identifier: NCT02208869) [7]. As of now, most of the genetic studies in Russian patients with FH have been performed as part of research programs before the era of next generation sequencing (NGS). Hence, the amount of publications is extremely limited. At the same time, a proper genetic study is essential to confirm FH. The evaluation of additional genes associated with dyslipidemia and premature atherosclerosis may help to identify additional genetic factors which could modify the phenotype [8].

The aim of this study was to investigate the presence of pathogenic mutations in a cohort of patients with definite or probable diagnosis of FH by using targeted NGS.

## 2. Materials and Methods

### 2.1. Subjects

The study included 52 unrelated patients with definite or probable FH based on the Dutch lipid clinic network criteria (DLCN score ≥ 6) [3]. It was a cohort of patients originating from different regions of Russia who were currently living in Moscow and previously underwent a medical examination at the National Medical Research Center of Cardiology (NMRCC), Moscow, Russia. Signed informed consent was necessary to participate in the study. Men and women aged 18–75 years with total cholesterol (TC) ≥ 7.5 mmol/L or low-density lipoprotein cholesterol (LDL-C) ≥ 4.9 mmol/L were included. The exclusion criteria were hypothyroidism (thyroid-stimulating hormone ≥ 1.5 UNL), glycosylated hemoglobin > 9% and creatinine clearance < 30 mL/min. The lipid parameters measurements and carotid duplex ultrasound was performed as part of medical examination during the period of one year at the moment of selection for genetic study. The clinical information, current laboratory data and carotid arteries duplex ultrasound description were taken from clinical examination and medical records of laboratory and instrumental evaluation performed at NMRCC, Moscow, Russia. The study was approved by the ethics committee of the NMRCC.

### 2.2. Genetic Study

The blood samples were studied by massive parallel sequencing using a custom capture library. Coding exons and exon–intron boundaries of 63 genes (Table 1, Table S1) related to dyslipidemia and premature atherosclerosis were captured using a custom probe library (SureSelect Target Enrichment Kit for Illumina paired-end multiplexed sequencing method; Agilent Technologies, Santa Clara, CA, USA).

Sequencing was performed using the Illumina HiSeq 1500 platform (Illumina, San Diego, CA, USA) with 2 × 100 base pair read length following Illumina protocols. Bioinformatics analysis was performed by means of a custom pipeline that includes software as NovoAlign (Novocraft Technologies Sdn Bhd), SAMtools and BCFtools (Sanger Institute) for variant calling and genotyping and Annovar for variant annotation. This genetic test is aimed at identifying single nucleotide variants (SNVs) and small insertions/deletions up to 20 bp. Sanger sequencing was used to evaluate regions of low coverage and to confirm the genetic variants considered clinically relevant according to the patient’s phenotype. The analytical sensitivity and accuracy is greater than 99%. Copy number variations were also evaluated using a comparative depth-of-coverage strategy.

To establish the pathogenicity of the variants, we adopted a customized classification scheme based on the recommendations of the American College of Medical Genetics and Genomics (ACMG) (Table S2) [9]; information regarding frequency in different populations (1000 Genomes Project, Exome Variant Server, Exome Aggregation Consortium, Genome Aggregation Database [v2.1.1 version]) was considered. The final classification of each variant was agreed by consensus between two clinical cardiologists and genetics experts.

### 2.3. Laboratory Analysis

The TC, triglycerides (TG) and high-density lipoprotein cholesterol (HDL-C) serum levels were measured after 12-h fasting in NMRCC’s certified laboratory at the time of inclusion in the study. LDL-C was calculated using the Friedewald formula [10]. TG levels were <4.5 mmol/L in all patients. Lp(a) concentration was determined by enzyme-linked immunosorbent assay using monospecific polyclonal sheep anti-human-apo(a) antibodies as previously reported [11].

### 2.4. Duplex Scanning of Carotid Arteries

Carotid duplex ultrasound was performed using the Philips IU22 ultrasound system with 9–12 MHz linear array transducer in the NMRCC ultrasound laboratory. Atherosclerotic plaques were assessed in distal part of common carotid arteries (CCA) in anterior position, in bifurcation of CCA and in internal carotid arteries (ICA) from right and left side. Carotid plaques were defined as focal structures encroaching into the arterial lumen of at least 0.5 mm or 50% of surrounding intima–media thickness value or demonstrating a thickness greater than 1.5 mm as measured from the intima-lumen interface to the media–adventitia interface [12]. The carotid stenosis evaluation was made by using the ECST criteria [13]. The maximum percentage of arterial stenosis, summary percentage of arterial stenosis, and atherosclerotic plaques amount were evaluated. Atherosclerotic plaques amount was defined as the total amount of all plaques found in distal part of CCA, bifurcation of CCA and in ICA from right and left sides. Max% of stenosis was defined as the maximum stenosis noted. Summary% of stenosis was defined as the total amount of all stenosis found in distal part of CCA, bifurcation of CCA, and in ICA from right and left sides.

### 2.5. Statistical Analysis

Statistical analysis was performed with Statistica 6.0 (StatSoft Inc., Tulsa, OK, USA). Continuous variables are given as medians and interquartile ranges (lower and upper quartiles), Me(LQ-UQ). The characteristics between the groups were compared using the Mann–Whitney U test for continuous variables, and Fisher exact two-tailed and chi-squared tests for categorical data; *p* value < 0.05 was considered statistically significant. No Bonferroni corrections were made.

## 3. Results

### 3.1. Genetic Test Results

Likely pathogenic/pathogenic mutations, modifying factors, and protective factors were selected among other genetic variations while analyzing the results of NGS to prepare a clinical report for each patient. The information about the presence of these variants with their established clinical significance, associated max LDL-C/TC levels, data on cardiovascular disease (CVD), and a family history of death from myocardial infarction (MI) are given in Table 2.

Pathogenic/likely pathogenic mutations considered to be responsible for monogenic FH were identified in 25 out of the 52 of probands (48%): 24 with mutations in LDLR, 4 homozygous FH and 20 heterozygous FH (HeFH) carriers and 2 with a mutation in APOB (one of them also carried a pathogenic mutation in LDLR). There were 22 pathogenic/likely pathogenic mutations in LDLR: 12 missense, 3 frameshift, 3 deletions, 1 duplication and 3 splice-site mutations. Eight out of these 22 mutations in LDLR have not been previously described in the literature (Gly119Valfs*12, c.940 + 3_940 + 6delGAGT, Tyr489Asn, Lys581Gln, Ser586Pro, c.1846-3T > G, Glu714_Ile796del, c.2389 + 5G > C). Only three (Pro220_Asp221del, Cys329Tyr, Trp443Arg) out of the 22 LDLR mutations have been previously reported in FH patients from Russia. The most frequent was a well-known mutation in LDLR, Gly592Glu, a founder mutation in a subpopulation in northwest Greece [14], also reported in the US, Europe and Latin America, which was found in four probands. The 22 mutations identified in the LDLR gene with their associated LDL-C levels, functional studies, and previous publications are listed in Table 3; additional information about predicted functional effects can be found in Tables S3–S5. Two patients carried a well-known pathogenic variant in the APOB gene, Arg3527Gln, which has been identified in combination with Glu2566Lys (rs1801696), a rare polymorphism in this gene [15]. There were no mutations in the proprotein convertase subtilisin/kexin type 9 gene.

All four patients with clinical picture of homozygous FH (maximum TC range of 17.3–23.0 mmol/L, maximum LDL-C range of 15.2–21.0 mmol/L) had two LDLR heterozygous mutations (Table 2).

All seven carriers who were heterozygous for the Ile1891Met polymorphism (rs3798220) in the lipoprotein(a) (LPA) gene [16] had elevated lipoprotein(a) [Lp(a)] levels, of 117.8 (105.7–123.8) mg/dL, ranging between 92.7 and 213.0 mg/dL. Among non-carriers of Ile1891Met (or other variants in LPA to modify the phenotype), 16 patients had Lp(a) > 50 mg/dL, with median 71.1 (64.3–102.6) mg/dL. In addition, 12 probands were carriers of several polymorphisms (rs17602729, rs34526199, rs61752479) in the adenosine monophosphate deaminase 1 (AMPD1) gene [17], although they tolerated statins well. There were no dramatic adverse events on statin treatment; however, in 7 patients one statin was changed for another due to elevated transaminases, muscle pain or weakness (3 with and 4 without polymorphisms in AMPD1).

### 3.2. Clinical Data Analysis for Heterozygous FH

**Figure 1:**
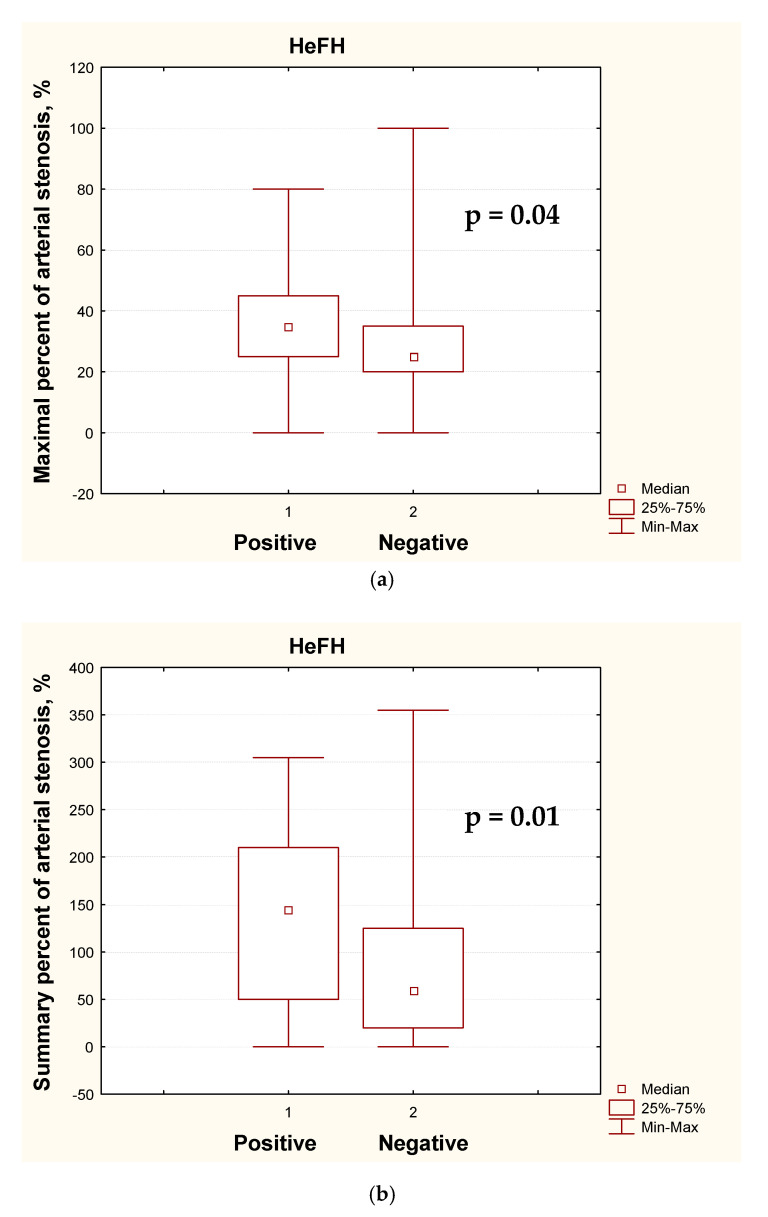

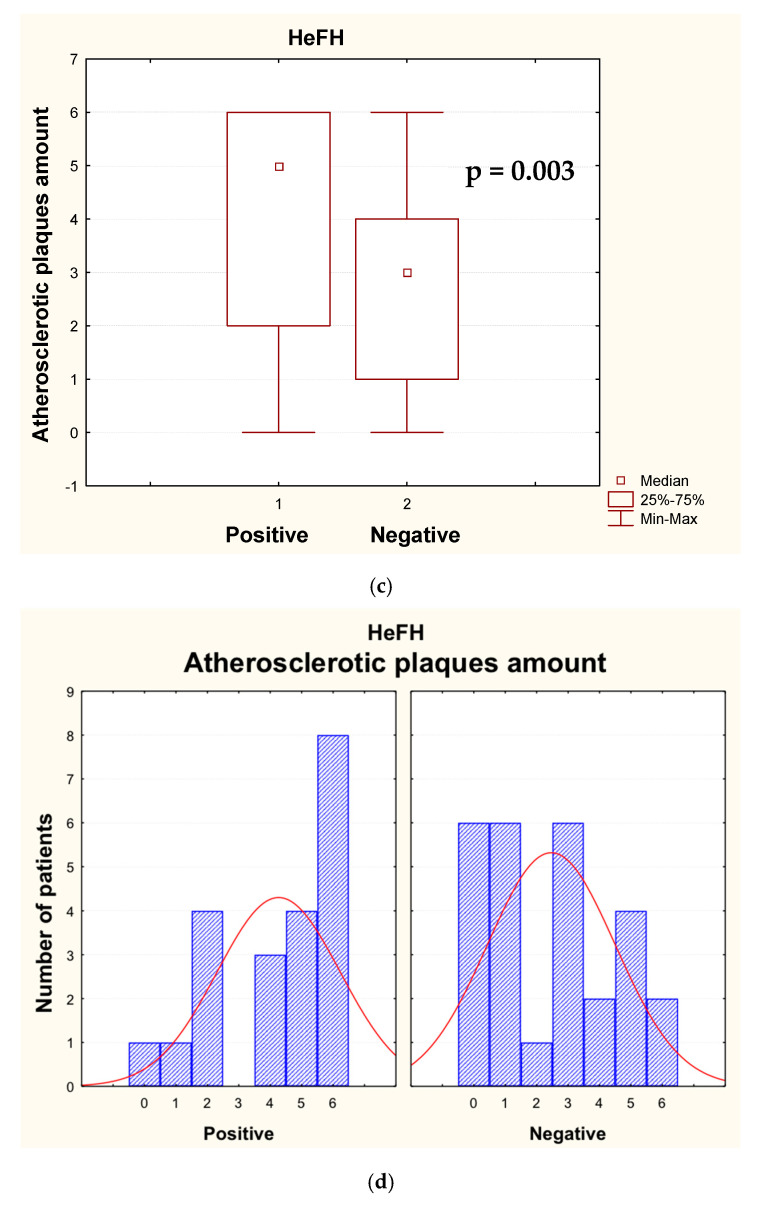
The carotid duplex ultrasound data in mutation-positive (1) and mutation-negative (2) patients with clinically established heterozygous familial hypercholesterolemia (HeFH): (**a**) maximal percent of arterial stenosis; (**b**) summary percent of arterial stenosis; (**c**,**d**) atherosclerotic plaques amount.

The comparison of clinical characteristics of mutation-positive and mutation-negative patients with HeFH is given in Table 4. Among patients with clinically established HeFH, the maximum TC and LDL-C levels were higher in those with positive than in those with negative genetic test results (*p* = 0.01 and *p* = 0.0004, respectively). However, current untreated median LDL-C levels were quite similar (8.9 mmol/L in positive and 7.9 mmol/L in mutation-negative cases, *p* = 0.04), as well as the number of patients who had suffered from MI, which did not significantly differ between the groups. Although mutation-negative patients had highly elevated TC and LDL-C levels, only a half of them had a positive family history of hypercholesterolemia in first-degree relatives. The mutation-positive patients had more severe atherosclerotic lesions in carotid arteries and a higher percentage of premature peripheral artery disease (*p* = 0.03), see Figure 1.

## 4. Discussion

With the development of new technologies, the impact of genetic studies for clinical practice is being widely evaluated. It became clear that the genetic cause of the disease is not always identifiable in patients with a clinical diagnosis of FH. On the other hand, in some patients, this genetic cause does not involve only one gene (polygenic cause of the disease, determined by the accumulation of common variants, single nucleotide polymorphisms [SNPs], which increase LDL-C) [18]. In our study, the disease-causing mutations, mostly in LDLR, have been identified only in 48% of patients with clinical diagnosis of FH, which is consistent with data received from other countries. At the same time there was a difference among mutation-positive and mutation-negative patients in clinical presentation of disease (Table 4).

There are convincing data confirming that the presence of a FH-related mutation leads to prolonged exposure to LDL-C and, therefore, significantly elevated cardiovascular (CV) risk [19]. The preclinical atherosclerosis, assessed by carotid intima–media thickness measurements and the coronary artery calcium score, was found to be more severe in patients with monogenic FH [20]. It was shown that among individuals with LDL-C ≥ 4.9 mmol/L, those with pathogenic mutations identified by sequencing three main FH genes (LDLR, APOB and PCSK9) have approximately 3.7-fold higher risk for coronary artery disease [21]. In genotyped HeFH patients registered in the Norwegian Cause of Death Registry during 1992–2013, CVD was the most common cause of death, with mean age of 64.5 years [22]. However, the number of patients in our study who had suffered from MI did not significantly differ depending on a presence of pathogenic mutations, for all patients with heterozygous FH, as well as when calculated for 50-year-olds or younger (*p* = 0.3). These data support the findings that other CV risk factors, besides FH, add significant impact in possibility of MI development [23]. The independent association of age, male sex, increased body mass index (BMI), hypertension, type 2 diabetes mellitus, previous use of tobacco and Lp(a) > 50 mg/dL with atherosclerotic CVD was confirmed in patients from the SAFEHEART Registry (Spanish Familial Hypercholesterolemia Cohort Study) with molecularly defined HeFH [24]. Recently, several risk scores (the Montreal-FH-SCORE, the SAFEHEART-RE risk equation) based on simple clinical predictors were found appropriate for CV risk assessment in patients with a genetically confirmed HeFH [25,26] Similarities in parameters such as arterial hypertension, smoking habit, BMI, HDL-C levels, age and gender were present between the groups of mutation-positive and mutation-negative patients in our study (Table S6).

The phenotype may also be modified by common variants associated with high LDL-C levels and CVD risk, which sometimes brings a visible variability within one family [27]. Among mutation-negative patients in our study, there were no significant differences in lipid levels depending on the presence of the APOE4 allele, showing the importance of particular combinations of SNPs for a prominent hypercholesterolemia.

## 5. Conclusions

Monogenic FH has been genetically confirmed in about half of the patients with definite or probable diagnosis of FH in our study, and the importance of a genetic study to determine the pathogenic cause of FH can be emphasized. Carriers of pathogenic mutations are more likely to have severe atherosclerotic lesions and tendon xanthomas due to prolonged exposure to LDL-C. Moreover, the autosomal-dominant type of inheritance predicts the signs of disease in relatives, making genetic cascade family screening reasonable for a timely identification of individuals at high CV risk [28]. At the same time, the importance of other CV risk factors should not be underestimated in patients with FH.

## Figures and Tables

**Table 1:** List of 63 evaluated genes related to dyslipidemia and premature atherosclerosis.

Associated Phenotypes	Genes
Monogenic dyslipidemias	ABCA1, ABCG1, ABCG5, ABCG8, ANGPTL3, APOA1, APOA5, APOB, APOC2, APOC3, APOE, CETP, GPD1, GPIHBP1, LCAT, LDLR, LDLRAP1, LIPA, LIPC, LMF1, LPA, LPL, LRP6, MEF2A, MTTP, MYLIP, PCSK9, PLTP, SAR1B, SCARB1, SLC25A40
Other inherited conditions related to dyslipidemia and premature atherosclerosis	AGPAT2, AKT2, AMPD1, BLK, BSCL2, CAV1, CEL, CIDEC, COQ2, CPT2, CYP2D6, GCK, HNF1A, HNF1B, HNF4A, INS, INSR, KLF11, LEP, LMNA, NEUROD1, NPC1L1, PAX4, PDX1, PLIN1, PNPLA2, PPARA, PPARG, PTRF, PYGM, SLC22A8, ZMPSTE24

**Table 2:** Genetic tests results, disease phenotype and family history of death from myocardial infarction in 52 probands.

Proband’s ID	Genetic Data			Clinical Data						Family History of Death from MI	
	Mutations		Modifying Factors ^2^	Max LDL-C/TC Levels, mmol/L ^10^	Lp(a) Levels, mg/dL	HDL-C Levels, mmol/L	Age, Years	Sex	Cardiovascular Disease	First-Degree Relatives	Second-Degree Relatives
	*LDLR*	*APOB*									
1	-	Arg3527Gln	*APOB* (Glu2566Lys) ^3^	6.19/8.60	1.6	1.78	48	F	-	-	-
2	Gly592Glu	-	-	10.21/11.94 ^9^	-	1.35	65	F	PAD	-	-
4	c.940 + 3_940 + 6delGAGT	-	*APOE* (Cys130Arg) ^3^ *APOA5* (Ser19Trp) ^4^	9.17/11.89	68.1	1.37	42	F	-	-	Maternal uncle at 45yr, maternal grandmother at 59yr, maternal uncle SD at 57yr
6	-	-	*LPL* (Asn318Ser) ^4^	7.60/9.60	7.5	1.2	56	F	PCI at 51yr	-	-
7	Cys329Tyr	-	-	10.00/12.00 ^9^	13.2	1.27	51	M	PAD	-	-
8	Gly592Glu	-	*APOE* (Cys130Arg) ^3^ *LDLR* (Val827Ile) ^8^	11.50/14.00	31	1.44	55	M	CABG at 48yr, PCI at 50yr; PAD	-	-
9	-	-		6.06/8.00	55.4	1	67	F	-	-	-
10	-	-	*LPA* (Ile1891Met) ^5^	8.43/11.11	119.2	1.78	61	F	-	-	-
11	-	-	-	7.11/9.21	106.4	1.09	42	M	-	-	-
12	-	-	-	7.87/10.14	11.7	1.38	42	M	-	-	-
13	-	-	-	9.26/11.43	54.9	1.35	45	M	-	-	-
14	-	-	*APOE* (Cys130Arg) ^3^	9.4/11.50	70.9	0.76	43	M	-	-	-
16	Cys352Arg	-	*APOE* (Cys130Arg) ^3^ *LPA* (Ile1891Met) ^5^	8.59/10.13	92.7	1	19	M	-	-	Paternal uncles at 45yr and 63yr
17	-	-	-	10.85/14.25	91.3	1.22	62	F	CAD at 51yr, MI and CABG at 62yr	Mother at 38yr	-
18	Pro106_Val395dup	-	*APOE* (Cys130Arg) ^3^ *APOA5* (Ser19Trp) ^4^	8.17/9.94 ^9^	8.8	0.81	68	M	MI at 51yr, CABG at 61yr	Father at 67yr	-
19	Val806Glyfs*11	Arg3527Gln	*APOB* (Glu2566Lys) ^3^ *PCSK9* (Arg93Cys) ^7^	9.36/12.00	143.3	2.02	61	F	PCI at 48yr; PAD	Father at 69yr	Paternal uncle at 46yr
21	c.1846-3T > G	-	-	8.94/10.57	98.7	1.02	55	M	CAD at 46yr, CABG at 50yr; PAD	-	Maternal uncle at 54yr
22	Ser586Pro	-	*APOE* (Cys130Arg) ^3^	9.68/12.00	4.5	1.3	46	M	CAD at 36yr, MI and CABG at 37yr; PAD	Mother at 58yr	Two maternal uncles before 40yr
23	c.2389 + 5G > C	-	*LPA* (Ile1891Met) ^5^	13.11/15.67 ^9^	117.8	1.54	50	F	-	-	Maternal grandfather at 52yr
24		-	*APOE* (Cys130Arg) ^3^	7.76 / 9.96	5	1.92	42	F	-	-	-
25	Ser177Leu, Cys352Arg	-		17.63/19.00	190.3	0.79	32	F	CAD at 20yr, MI at 30yr; PAD	-	-
28	Cys329Tyr, Gly592Glu	-	*APOA5* (Ser19Trp) ^4^	17.35/19.00	-	0.98	39	F	CAD at 36yr	-	-
29	Arg416Trp, c.940 + 3_940 + 6delGAGT	-	-	15.15/17.25	55.8	0.94	31	F	MI at 15yr, CABG at 26yr; PAD	-	-
30	Pro220_Asp221del	-	-	7.00/9.00	6.5	0.95	46	M	MI at 24yr, PCI at 26yr	Mother at 62yr	-
33	Gln739*	-	-	7.00/9.10 ^9^	79.8	0.8	36	M	MI and PCI at 36yr	-	-
34	Gly119Valfs*12	-	*APOE* (Cys130Arg) ^3^ *ABCA1* (Lys776Asn) ^6^	11.00/13.00	69.7	0.96	69	F	MI at 52yr, PCI at 66yr	Mother at 59yr, son SD at 25yr	Maternal mother at 64yr, maternal aunt at 69yr
35	-	-	-	7.00/9.00	3.3	0.85	47	M	CAD at 39yr, PCI at 40yr, MI at 46yr; PAD	-	-
36	Gly545Arg	-	-	11.90/13.79	25	1.01	53	F	CAD at 47yr, PCI at 52yr; PAD	-	-
37	Tyr489Asn	-	*APOA5* (Ser19Trp) ^4^	14.82/17.61	-	1.1	37	M	MI at 28yr, PCI at 29yr	-	Maternal aunt at 33yr, maternal grandfather at 33yr
38	Glu714_Ile796del, Trp443Arg	-	-	21.00/23.00	134	0.82	27	F	MI at 21yr, CABG at 23yr; PAD	-	Maternal grandmother at 60yr
39	Lys581Gln	-	-	11.18/12.80	-	0.85	42	F	-	-	
40	Leu64_Pro105delinsSer, Pro181Leu	-	-	8.83/10.83	-	1.14	45	F	MI at 40yr, CABG at 43yr	-	Paternal uncle at 57yr
41	-	-	-	6.70/9.38	65.6	1.06	60	M	CAD at 53yr, MI at 58yr, CABG at 59yr; PAD	-	-
42	-	-	-	7.53/9.68 ^9^	63	1.3	70	F	CAD at 48yr, MI at 51yr; PAD	Father at 61yr	-
43	-	-	-	6.08/9.14	8.8	1.98	59	F	CAD at 56yr	-	-
44	-	-	*LPA* (Ile1891Met) ^5^	6.90/9.05	105.7	1.64	61	F		-	-
45	Glu308Lys	-	*APOE* (Cys130Arg) ^3^ *LPA* (Ile1891Met) ^5^	8.30/10.30	123.8	1.45	46	M	CAD at 44yr, CABG at 45yr	-	-
46	-	-	*APOE* (Cys130Arg) ^3^ *LPA* (Ile1891Met) ^5^	8.00/10.00	116.3	1.53	65	F	-	-	-
47	Ser586Pro	-	*LPA* (Ile1891Met) ^5^	7.84/9.75	213	0.82	34	M	CAD at 31yr, MI and PCI at 33yr	Father SD at 60yr	-
48	-	-	-	5.55/8.00 ^9^	21.3	1.66	65	M	MI and PCI at 59yr; PAD	Father SD at 48yr	-
49	-	-	*APOE* (Cys130Arg) ^3^	5.75/7.95	7	1.37	27	M	MI and PCI at 21yr	-	Maternal grandfather at 62yr
50	-	-	*LIPC* (c.-57_88del) ^4^	9.00/11.00	16.6	1.17	68	F	-	-	-
51	Gly592Glu	-	-	11.84/14.17	7.5	1.13	59	F	-	-	-
52	-	-	-	14.67/17.00	5.2	1.65	32	M	-	-	-
53	-	-	*APOE* (Cys130Arg) ^3^ *LPL* (Ser474*)	7.90/9.90	2.9	1.22	56	M	CAD at 54yr	-	-
54	-	-	*APOE* (Cys130Arg) ^3^	8.61/11.26	2.6	0.68	46	M	-	-	-
55	-	-	*APOA5* (Ser19Trp) ^4^	6.1/8.64	4	1.19	69	M	-	-	-
56	-	-		6.92/9.60	9.9	2.24	55	F	CAD	-	-
57	-	-	*APOE* (Cys130Arg) ^3^	5.98/8.00	71.2	1.14	63	M	CAD at 38yr, MI and CABG at 50yr	Father at 48yr	-
58	-	-	*APOE* (Cys130Arg ^1^) ^3^	8.00/10.10	12.1	1.66	53	F	-	-	-
59	-	-	*APOE* (Cys130Arg) ^3^ *LPL* (Asn318Ser) ^4^	5.88/8.24	2.9	1.23	44	F	-	-	-
60	-	-	*APOA5* (Ser19Trp) ^4^	8.9/14.00 ^9^	-	1.4	44	M	MI at 38yr, PCI at 39yr; PAD	Father at 49yr	Paternal grandfather at 53yr

ABCA1, ATP-binding cassette sub-family A member 1; APOA5, Apolipoprotein A5; APOB, apolipoprotein B; APOE, apolipoprotein E; CABG, coronary artery bypass graft surgery; CAD, coronary artery disease; F, female; HDL-C, high-density lipoprotein cholesterol; ID, identifier; LDL-C, low-density lipoprotein cholesterol; LDLR, low density lipoprotein receptor; LIPC, hepatic triacylglycerol lipase; LPA, apolipoprotein(a); Lp(a), lipoprotein(a); LPL, lipoprotein lipase; M, male; MI, myocardial infarction; PAD, peripheral arterial disease; PCI, percutaneous coronary intervention; PCSK9, proprotein convertase subtilisin/kexin type 9; SD, sudden death; TC, total cholesterol; yr, year. ^1^ Homozygous. The unmarked variants are heterozygous. ^2^ Polymorphisms previously associated with higher ^3^ LDL-C, ^4^ TG or ^5^ Lp(a) levels, or ^6^ elevated ischemic heart disease risk. ^7^ Polymorphism in the Japanese population, previously associated with low LDL-C levels and decreased risk of atherosclerotic CVD, and considered to be a protective factor. ^8^ Rare variant of unknown clinical significance previously identified in patients with FH. ^9^ On statins. ^10^ LDL-C includes also Lp(a) cholesterol values.

**Table 3:** List of 22 mutations identified in the *LDLR* gene with their associated low-density lipoprotein cholesterol (LDL-C)/total cholesterol (TC) levels, functional studies and previous publications.

N	*LDLR* Variant	Exon/Intron	c.DNA	Number of Pts	Associated ^1^ Max LDL-C/TC Levels, mmol/l	Previously Published in Publications	ExAC Database Frequency	GnomAD Allele Frequencies	Familial Cosegregation	Functional Studies	Number of Carriers/Families	Countries (Previously Published)	Pathogenicity
1	Leu64_Pro105delinsSer	Ex3	c.191_313del	1	–	6	0	0	–	+	NA	France, the Netherlands	+++
2	Pro106_Val395dup	DupEx4–8	c.*11173514_*11173515	1	8.17/9.94 ^2,3^	3	0	0	–	–	NA	Poland, the Czech Republic, the Netherlands	+++
3	Gly119Valfs*12	Ex4	c.355_356insTTCC	1	11.00/13.00 ^2,3^	0	0	0	–	–	0	–	+++
4	Ser177Leu, FH Puerto Rico	Ex4	c.530C > T	1	–	36	1/121078	4/251308	+	+	59/33	Europe, Latin America, USA	+++
5	Pro181Leu	Ex4 ^4^	c.542C > T	1	–	1	0	2/251308	–	+	1/1	Brazil	+
6	Pro220_Asp221del	Ex4	c.658_663delCCCGAC	1	7.0/9.0	1	0	0	–	+	1/1	Russia	+++
7	Glu308Lys	Ex6	c.922G > A	1	8.3/10.3 ^2^	3	0	0	–	–	NA	Poland, the Netherlands	++
8	c.940 + 3_940 + 6delGAGT	Int6	c.940 + 3_940 + 6delGAGT	2	9.17/11.89 ^2^	0	0	0	–	–	0	–	+
9	Cys329Tyr	Ex7 ^4^	c.986G > A	2	10.00/12.00 ^3^	15	3/120592	7/282402	+	–	53/38	Asia (Taiwan, Philippines, China) and Russia	+++
10	Cys352Arg	Ex7 ^4^	c.1054 T > C	2	8.59/10.13 ^2^	4	0	0	–	–	NA	Austria	++
11	Arg416Trp	Ex9	c.1246C > T	1	–	42	3/120616	6/251158	+	+	NA	Europe (Spain, Norway, the UK, Germany, Austria, Czech Republic), Canada, Asia (Japan, Taiwan)	+++
12	Trp443Arg	Ex9 ^4^	c.1327 T > C	1	–	1	1/120926	1/251246	–	–	2/2	Russia	++
13	Tyr489Asn	Ex10 ^4^	c.1465 T > A	1	14.82/17.61	0	0	0	–	–	0	–	+
14	Gly545Arg	Ex11 ^4^	c.1633G > A	1	11.9/13.79	5	0	0	–	–	5/5	Brazil, France, Korea	++
15	Lys581Gln	Ex12 ^4^	c.1741A > C	1	11.18/12.80	0	0	0	–	–	0	–	+
16	Ser586Pro	Ex12	c.1756 T > C	2	9.68/12.00 ^2^ 7.84/9.39	0	0	0	–	–	0	–	+
17	Gly592Glu, FH Sicily, FH Foggia-1, FH Naples4	Ex12	c.1775G>A	4	10.21/11.94 ^3^ 11.50/14.00 ^2^ 11.84/14.17	41	6/121408	16/282866	+	+	31/27	The most common cause of FH in north-western Greece (34% of cases); USA, Europe, Latin America	+++
18	c.1846-3T > G	Int12	c.1846-3T > G	1	8.94/10.57	0	0	0	–	–	0	–	+
19	Glu714_Ile796del	Int14–Int16	c.2141-966_2390-330del	1	–	0	0	0	+	+	11/2	Japan, Brazil	+++
20	Gln739*	Ex15	c.2215C > T	1	7.0/9.1 ^3^	9	0	0	–	–	5/5	Italy, Mexico, Asia (Japan, Taiwan)	+++
21	c.2389 + 5G > C	Int16 ^4^	c.2389 + 5G > C	1	13.11/15.67 ^3^	0	0	0	–	–	0	–	++
22	Val806Glyfs*11	Ex17	c.2416_2417insG	1	–	17	5/121318	6/251326	+	–	20/10	USA, Europe, Middle East, Latin America, Japan	+++

c.DNA, coding DNA; ExAC, Exome Aggregation Consortium; FH, familial hypercholesterolemia; gnomAD, Genome Aggregation Database (v2.1.1 version); LDL-C, low-density lipoprotein cholesterol; LDLR, low density lipoprotein receptor; N, mutation number; NA, not available; pts, patients; TC, total cholesterol; +++, Pathogenic; ++, Very likely pathogenic; +, Likely pathogenic. ^1^ The max LDL-C/TC levels in a single-mutation heterozygous carriers (carriers of more than one mutation in *LDLR* are excluded). ^2^ Heterozygous for ApoE4. ^3^ On statins. ^4^ Other variants in the same position were previously reported in association with FH.

**Table 4:** Clinical characteristics of patients with heterozygous familial hypercholesterolemia.

Clinical Characteristics		Mutation		*p*
		Positive (*n* = 21)	Negative (*n* = 27)	
**Clinical data**				
Men/Women		11/10	14/13	NS
Mean age, years		48 (42–55)	56 (44–63)	NS
BMI, kg/m^2^		27.4 (24.1–30.8)	28.4 (24.5–31.1)	NS
Smokers: Present/Gave up		29%/24%	22%/33%	NS
Arterial hypertension		67%	56%	NS
**Family history**				
Premature CAD		52%	63%	NS
Tendon xanthomas		14%	0%	0.08
Hypercholesterolemia		86%	48%	0.007
**Personal history**				
Tendon xanthomas		33%	11%	0.06
Premature CAD/CAD		62%/62%	37%/44%	0.08/NS
Myocardial infarction		38%	33%	NS
Age of MI		36.5 (30.5–45.5)	50.5 (42.0–58.5)	NS
PCI		38%	19%	NS
CABG		29%	11%	NS
Premature PAD/PAD		33%/33%	7%/19%	0.03/NS
Ischemic stroke		5%	4%	NS
**Laboratory data**				
Max TC, mmol/L		11.9 (10.1–13.0)	9.6 (9.0–11.0)	0.01
Max LDL-C, mmol/L		9.4 (8.3–11.2)	7.6 (6.6–8.6)	0.0004
Current lipid levels, mmol/L	TC	10.3 (9.1–12.0)	9.7 (8.1–11.1)	NS
	LDL-C	8.6 (7.0–10.2)	7.6 (6.1–8.6)	0.07
	HDL-C	1.1 (1.0–1.4)	1.3 (1.1–1.7)	NS
	TG	1.6 (1.2–2.3)	1.9 (1.7–2.1)	NS
Current LDL-C without treatment, mmol/L		8.9 (8.2–11.5) (*n* = 12)	7.9 (6.7–8.6) (*n* = 21)	0.04
Lp(a), mg/dL		68.1 (8.8–98.7) (*n* = 17)	14.35 (5.2–70.9) (*n* = 26)	NS
**Duplex ultrasound of carotid arteries data**				
Max% of stenosis ^1^		35 (25–45)	25 (20–35)	0.04
Summary% of stenosis ^2^		145 (50–210)	60 (20–125)	0.01
Atherosclerotic plaques amount ^3^		5 (2–6)	3 (1–4)	0.003

BMI, body mass index; CABG, coronary artery bypass graft surgery; CAD, coronary artery disease; HDL-C, high-density lipoprotein cholesterol; LDL-C, low-density lipoprotein cholesterol; Lp(a), lipoprotein(a); MI, myocardial infarction; PAD, peripheral arterial disease; PCI, percutaneous coronary intervention; TC, total cholesterol; TG, triglyceride. NS, *p* ≥ 0.1. ^1^ One maximal stenosis is chosen. ^2^ Total amount of all stenosis found in distal part of common carotid artery, bifurcation of common carotid artery and in internal carotid artery from right and left sides. ^3^ Total amount of all plaques found in distal part of common carotid artery, bifurcation of common carotid artery and in internal carotid artery from right and left sides.

## Supplementary Materials

The following are available online at https://www.mdpi.com/2308-3425/7/2/16/s1, Table S1. List of 63 evaluated genes related to dyslipidemia and premature atherosclerosis with associated phenotypes. Table S2: Customized classification scheme based on the recommendations of the ACMG used for establishing the pathogenicity of the identified variants, Table S3: List of mutations identified in the LDLR gene with nomenclature and their predicted functional effects on the protein, Table S4: Mutation effect prediction algorithms—Missense mutations in LDLR, Table S5: Mutation effect prediction algorithms—Splice-site mutations in LDLR, Table S6: Cardiovascular risk factors and presence of myocardial infarction in patients with heterozygous familial hypercholesterolemia.
