# Supplementary material for: Verification of Underlying Genetic Cause in a Cohort of Russian Patients with Familial Hypercholesterolemia Using Targeted Next Generation Sequencing
Source: J Cardiovasc Dev Dis. 2020 May 14;7(2):16. doi: 10.3390/jcdd7020016 (PMC7345545; doi:10.3390/jcdd7020016)
Supplement: Supplementary file 1 [file jcdd-07-00016-s001.pdf]

Supplementary Materials

Table S1. List of 63 evaluated genes related to dyslipidemia and premature atherosclerosis with associated phenotypes.

Table S2. Customized classification scheme based on the recommendations of the ACMG used for establishing the pathogenicity of the identified variants.

Table S3. List of mutations identified in the LDLR gene with nomenclature and their predicted functional effects on the protein.

Table S4. Mutation effect prediction algorithms – missense mutations in LDLR.

Table S5. Mutation effect prediction algorithms – splice-site mutations in LDLR.

Table S6. Cardiovascular risk factors and presence of myocardial infarction in patients with heterozygous familial hypercholesterolemia.

Table S1. List of 63 evaluated genes related to dyslipidemia and premature atherosclerosis with associated phenotypes.

Associated phenotypes	Gene	Encoded protein	Associated phenotype
Monogenic dyslipidemias	ABCA1	ATP-binding cassette sub-family A member 1	Tangier disease (AR)<br>HDL deficiency type 2 (AD)
	ABCG1	ATP-binding cassette sub-family G member 1	Hypoalphalipoproteinemia and increased risk of cardiovascular events
	ABCG5	ATP-binding cassette sub-family G member 5	Sitosterolemia (AR)
	ABCG8	ATP-binding cassette sub-family G member 8	Sitosterolemia (AR)
	ANGPTL3	Angiopoietin-related protein 3	Familial hypobetalipoproteinemia, type 2 (AR) - loss-of-function mutations<br>Predisposition to atherosclerosis due to increased LPL inhibition - gain-of-function mutations
	APOA1	Apolipoprotein A-I	HDL deficiencies, including Tangier disease<br>Systemic non-neuropathic amyloidosis
	APOA5	Apolipoprotein A-V	Elevated TG levels
	APOB	Apolipoprotein B 100 or Apolipoprotein B 48	Familial defective apoB-100 (AD) – mutations within the apoB-100 receptor-binding domain<br>Familial hypobetalipoproteinemia or abetalipoproteinemia (AD) - loss-of-function mutations
	APOC2	Apolipoprotein C-II	Hyperlipoproteinemia type IB
	APOC3	Apolipoprotein C-III	Associated with decreased plasma triglyceride levels and reduced risk of CAD – loss-of-function mutations
	APOE	Apolipoprotein E	Familial dysbetalipoproteinemia, type III hyperlipoproteinemia (HLP III), and familial hypercholesterolemia
	CETP	Cholesteryl ester transfer protein	Hyperalphalipoproteinemia (AD)
	GPD1	Glycerol-3-phosphate dehydrogenase [NAD(+)], cytoplasmic	Transient infantile hypertriglyceridemia (AR)
	GPIHBP1	Glycosylphosphatidylinositol-anchored high density lipoprotein-binding protein 1	Hyperlipoproteinemia, type 1D (AR)
	LCAT	Phosphatidylcholine-sterol acyltransferase	Fish-eye disease (AR),<br>LCAT deficiency (also known as Norum disease) (AR)
	LDLR	Low density lipoprotein receptor	Familial hypercholesterolemia (AD)
	LDLRAP1	low density lipoprotein receptor adaptor protein 1	Autosomal recessive hypercholesterolemia (AR)
	LIPA	Lysosomal acid lipase/cholesteryl ester hydrolase	Lysosomal acid lipase deficiency (AR):<br>Wolman disease and cholesteryl ester storage disease
	LIPC	Hepatic triacylglycerol lipase	Hepatic lipase deficiency (AR)

	LMF1	Lipase maturation factor 1	Combined lipase deficiency (AR)
	LPA	Apolipoprotein(a)	Lipoprotein(a) deficiency - null truncating mutations<br>Elevated Lp(a) levels - SNPs
	LPL	Lipoprotein lipase	Chylomicronemia syndrome, also known as Fredrickson hyperlipidemia type 1 or familial lipoprotein lipase (LPL) deficiency (AR)
	LRP6	Low-density lipoprotein receptor-related protein 6	Early coronary disease and metabolic syndrome (AD)
	MEF2A	Myocyte-specific enhancer factor 2A	Unproved relation to ischemic heart disease
	MTTP	Microsomal triglyceride transfer protein large subunit	Abetalipoproteinemia (AR)<br><br>SNPs: related to decreased serum levels of apoB containing lipoproteins (LDL-C and non-HDL-C) and contribution to development of non-alcoholic fatty liver disease
	MYLIP	E3 ubiquitin-protein ligase MYLIP	Proposed relation to LDL-C levels
	PCSK9	Proprotein convertase subtilisin/kexin type 9	Familial hypercholesterolemia, type 3 - gain of function mutations<br><br>LDL levels decrease - loss-of-function mutations
	PLTP	Phospholipid transfer protein	Proposed relation to HDL-C levels
	SAR1B	GTP-binding protein SAR1b	Chylomicron retention disease, also known as Anderson disease (AR)
	SCARB1	Scavenger receptor class B member 1	Increased level of plasma HDL-C with possible increased risk of CAD
	SLC25A40	Solute carrier family 25 member 40	Proposed relation to Familial hypertriglyceridemia
Other inherited conditions related to dyslipidemia and premature atherosclerosis	AGPAT2	1-acyl-sn-glycerol-3-phosphate acyltransferase beta	Generalized lipodystrophy type 1 (CGL1), or Berardinelli-Seip syndrome (AR)
	AKT2	RAC-beta serine/threonine-protein kinase	Diabetes mellitus, type 2 (AD),<br>Hypoinsulinemic hypoglycemia with hemihypertrophy (AD)
	AMPD1	adenosine monophosphate deaminase 1 (myoadenylate deaminase)	Adenosine monophosphate deaminase deficiency type 1 (AMPD1 deficiency), also referred to as myoadenylate deaminase deficiency (MMDD), with symptoms of metabolic myopathy (AR)<br>Presumed increased risk of drug-induced myopathy
	BLK	Tyrosine-protein kinase Blk	Maturity-onset diabetes of the young type 11 (AD)
	BSCL2	Seipin	Congenital generalized lipodystrophy type 2 or Berardinelli-Seip syndrome (AR)
	CAV1	Caveolin-1	Proposed relation to lipodystrophy (AD?)
	CAVIN1 (PTRF)	Polymerase I and transcript release factor, also known as Caveolae associated protein 1	Congenital generalized lipodystrophy type 4 (AR), with muscular dystrophy and cardiac conduction anomalies
	CEL	Bile salt-activated lipase	Maturity-onset diabetes of the young, type 8 (AD)
	CIDEC	Cell death activator CIDE-3	Insulin-resistant diabetes and partial lipodystrophy type 5 (AR)
	COQ2	4-hydroxybenzoate polyprenyltransferase, mitochondrial	Primary CoQ10 deficiency (AR)
	CPT2	Carnitine O-palmitoyltransferase 2, mitochondrial	Carnitine palmitoyltransferase type 2 deficiency (AR?)
	CYP2D6	Cytochrome P450 2D6	Alteration of the CYP2D6 enzyme function (ultra rapid, rapid, intermediate, and

			poor metabolizers) – mutations /polymorphisms
	GCK	Glucokinase	Non-insulin dependent diabetes mellitus, maturity-onset diabetes of the young type 2 (AD), Persistent neonatal diabetes (AR), Persistent hyperinsulinemic hypoglycemia of infancy
	HNF1A	Hepatocyte nuclear factor 1-alpha	Maturity-onset diabetes of the young subtype HNF1A, previously MODY3 (AD)
	HNF1B	Hepatocyte nuclear factor 1-beta	Renal cysts and diabetes syndrome (AD?),<br>Noninsulin-dependent diabetes mellitus (AD?)
	HNF4A	Hepatocyte nuclear factor 4-alpha	Maturity-onset diabetes of the young type 1
	INS	Insulin	Diabetes mellitus, insulin-dependent, 2 (AD?)<br>Diabetes mellitus, permanent neonatal (AD?)<br>Hyperproinsulinemia (AD?)<br>Maturity-onset diabetes of the young, type 10 (AD?)
	INSR	Insulin receptor	Inherited syndromes of extreme severe insulin resistance, including type A insulin resistance syndrome, Donohue syndrome and Rabson-Mendenhall syndrome
	KLF11	Krueppel-like factor 11	Maturity-onset diabetes of the young type 7 (AD)
	LEP	Leptin	Morbid obesity due to leptin deficiency (AR?)
	LMNA	Prelamin-A/C	Laminopathies (AD), including dilated cardiomyopathy, frequently associated with conduction defects (AV block, AF with slow ventricular response), muscular dystrophies (Emery-Dreifuss, limb girdle), familial lipodystrophy, Charcot-Marie-Tooth disease, and Hutchinson-Gilford progeria<br>Muscular dystrophies (AR)
	NEUROD1	Neurogenic differentiation factor 1	Maturity-onset diabetes of the young 6<br>Diabetes mellitus, noninsulin-dependent (AD?)
	NPC1L1	Niemann-Pick C1-like 1 protein	Association with plasma total and LDL-C levels and CAD risk:<br>reduced plasma LDL-C levels and a reduced risk of CAD - loss-of-function variants (AD)<br>higher LDL-C level and risk of CVD events - gain-of-function variants (AR)<br>Non-response to ezetimibe treatment (proposed)
	PAX4	Paired box protein Pax-4	Diabetes mellitus, type 2 (AD?)<br>Maturity-onset diabetes of the young, type 9
	PDX1	Pancreas/duodenum homeobox protein 1	Pancreatic agenesis (AR)<br>Maturity onset diabetes of the young type 4 (AD)<br>Susceptibility to diabetes mellitus type 2 (AD)
	PLIN1	Perilipin-1	Familial partial lipodystrophy 4 - loss-of-function mutations (AD)
	PNPLA2	Patatin-like phospholipase domain-containing protein 2	Neutral lipid storage disease with myopathy (AR)

	PPARA	Peroxisome proliferator-activated receptor alpha	Susceptibility to hyperapobetalipoproteinemia
	PPARG	Peroxisome proliferator-activated receptor gamma	Familial partial lipodystrophy type 3 (AD?)<br>Severe obesity (AD?, AR?, multifactorial)
	PYGM	Glycogen phosphorylase, muscle form	Glycogen storage disease type V, also known as McArdle disease (AR)
	SLC22A8	Solute carrier family 22 member 8	A candidate gene to evaluate drugs pharmacology, particularly of statins
	ZMPSTE24	CAAX prenyl protease 1 homolog	Mandibuloacral dysplasia, restrictive dermopathy

AD, autosomal dominant; AR, autosomal recessive; CAD, coronary artery disease; CVD, cardiovascular disease; HDL-C, high-density lipoprotein cholesterol; LDL-C, low-density lipoprotein cholesterol; Lp(a), lipoprotein(a); SNPs, single nucleotide polymorphisms; TG, triglyceride.

Table S2. Customized classification scheme based on the recommendations of the ACMG [1] used for establishing the pathogenicity of the identified variants.

Classification	Major Criteria	Supporting Criteria
PATHOGENIC OR DISEASE-CAUSING [+ + +]	1. Widely reported variant with conclusive evidence of a genotype-phenotype association and with consensus about its pathogenicity.<br>2. Demonstrated cosegregation with a phenotype (> 10 meioses).<br>3. Cosegregation in at least 2 families (≤ 10 meioses), or present in at least 5 probands with the same phenotype, and meeting at least 2 supporting criteria:	A. Protein-truncating variant in a gene where loss of function is a proven pathogenic mechanism.<br>B. Functional studies that support pathogenicity.<br>C. *De novo* presentation in the setting of a novel disease in the family (maternity and paternity confirmed).<br>D. Missense variant that generates the same amino-acid change as a previously reported pathogenic variant.<br>E. Variant with very low frequency/absent in the control population (MAF < 0.001%).
VERY LIKELY TO BE PATHOGENIC OR DISEASE-CAUSING [+ +]	1. Protein-truncating variant in a gene where loss of function is a proven pathogenic mechanism that explains the patient's phenotype, and that meets at least 1 supporting criterion:<br>2. Missense variant/in-frame insertion or deletion in a non-repetitive region of a gene with demonstrated genotype-phenotype association that explains the patient's disease, and that meets at least 2 supporting criteria:	A. Functional studies that support pathogenicity.<br>B. *De novo* presentation in the setting of a novel disease in the family (maternity and paternity confirmed).<br>C. Affecting a residue in which other pathogenic variants were previously identified (mutational hot spot); or variant located in a relevant functional domain or region of the protein.<br>D. Variant with very low allelic frequency/absent in the control population (MAF < 0.001%).<br>E. Probable cosegregation in at least one family, or various index cases, but that does not meet criteria for being considered pathogenic.
LIKELY TO BE PATHOGENIC OR DISEASE-CAUSING	1. Protein-truncating variant with very low frequency/absent in the control population (MAF < 0.001%) that affects a gene where loss of function is not	A. Variant with very low allelic frequency/absent in the control population (MAF < 0.001%).<br>B. *De novo* presentation in the setting of a novel disease

[+ ?]	an established pathogenic mechanism or that does not meet criteria to be considered pathogenic.<br>2. Intronic variant outside the consensus region of the gene for which the bioinformatics predictors agree that it would affect the splicing.<br>3. Missense variant/in-frame insertion or deletion in a non-repetitive region of a gene which does not meet criteria to be considered pathogenic/very likely to be pathogenic, but that meets at least 3 supporting criteria:	in the family (maternity and paternity unconfirmed).<br>C. Patient's phenotype or family history suggests that disease could be explained by mutations in the gene (gene with well-established phenotype-genotype association).<br>D. Bioinformatics predictors agree that it would be deleterious.<br>E. Located in a mutational hot spot, functional domain, or relevant region of the codified protein.<br>F. Reported in at least 2 unrelated individuals that presented the same phenotype.
UNKNOWN CLINICAL SIGNIFICANCE<br>[?]	1. Variants with contradictory information about their pathogenicity.<br>2. Variants that do not meet criteria for being included in another classification category.	
UNLIKELY TO BE PATHOGENIC OR DISEASE-CAUSING<br>[- ?]	1. Variant allele frequency in control populations is higher than the expected for disease or has a MAF > 0.05%.<br>2. Absence of variant cosegregation with the phenotype in at least 1 family.<br>3. Meeting at least 2 supporting criteria:	A. Missense variant in a gene where only variants causing protein truncation have shown association with disease.<br>B. Functional study showing that the variant does not affect the structure or function of the encoded protein.<br>C. Bioinformatics predictors agree that the variant would not alter the function of the protein (including splicing variants outside the consensus region of the gene).<br>D. In-frame insertions/deletions in a repetitive gene region without a known function.<br>E. Presence of the variant in homozygosis in control population.
NON-PATHOGENIC (NOT DISEASE-CAUSING)<br>[- -]	1. MAF > 5% in any of the control population databases.<br>2. Previously reported in the literature with well-established evidence of consensus about its non-disease-causing classification, and with no	A. Variant allele frequency in control populations is higher than expected for disease or has a MAF > 0.05%.<br>B. Absence of cosegregation of the variant with the

	contradictory data.<br>3. Absence of cosegregation with the disease in at least 2 reported families.<br>4. Meeting at least 2 supporting criteria:	phenotype in at least 1 family.<br>C. Functional study showing that the variant does not affect the structure or function of the encoded protein.<br>D. Presence of the variant in healthy unaffected subjects at an age at which the disease should be fully penetrant (variant must be in homozygosis in recessively inherited diseases, or in hemizygosis in X-linked diseases).

MAF, minor allele frequency.

[1] Richards S, Aziz N, Bale S, Bick D, Das S, Gastier-Foster J, et al.; ACMG Laboratory Quality Assurance Committee. Standards and guidelines for the interpretation of sequence variants: a joint consensus recommendation of the American College of Medical Genetics and Genomics and the Association for Molecular Pathology. Genet Med 2015 May;17(5):405-24.

Table S3. List of mutations identified in the *LDLR* gene with nomenclature and their predicted functional effects on the protein.

N	Exon/ Intron	Chr DNA level name	c.DNA	Protein / Alternative name	Position in *LDLR*	Predicted functional effect	Pathogenicity
1	Ex3	g.11213340_11213462del	c.191_313del	Leu64_Pro105delinsSer	a complete in-frame deletion of exon 3 of the *LDLR* gene and a loss of LDLR class A repeat 2, which is included in the N-terminal region of LDLR, responsible for ligand binding	a complete deletion of cysteine-rich class A repeat 2, which impairs LDL binding by up to 95% and does not impair the binding of beta-VLDL (Hobbs HH, et al., 1992) [1]	+++
2	DupEx4-8	g.22415506_22415507	c.*11173514_*11173515	Pro106_Val395dup	in-frame duplication of *LDLR* exons 4 to 8	would duplicate LDLR class A repeats 3-6 in the mature LDLR protein	+++
3	Ex4	**g.11215937_11215938insTTCC**	**c.355_356insTTCC**	**Gly119Valfs*12**	exon 4	a null-mutation that leads to the loss of many important domains, including part of the LDLR class A domains and the six LDLR class B domains	+++
4	Ex4	g.11216112C>T	c.530C>T	Ser177Leu / FH Puerto Rico	LDLR class A repeat 4	reduced LDL-uptake by LDLR in heterozygous (Thormaehlen AS, et al., 2015) [2] and < 2% LDLR activity in homozygous (Hobbs HH, et al., 1989) [3]	+++
5	Ex4	g.11216124C>T	c.542C>T	Pro181Leu	LDLR class A repeat 4	replacement of a single conserved amino acid in a cysteine-rich repeat (LDLR class A repeat 4) produces impaired LDL binding (Russell DW, et al., 1989) [4]	+
6	Ex4	g.11216240_11216245delCCCGAC	c.658_663delCCCGAC	Pro220_Asp221del	LDLR class A repeat 5	in-frame deletion of two amino acids located in the cysteine-rich class A repeat 5, which is included in the N-terminal region of LDLR, responsible for ligand binding (Hobbs HH, et al., 1992) [1]	+++
7	Ex6	g.11218172G>A	c.922G>A	Glu308Lys	LDLR class A repeat 7	belongs to the N-terminal LDLR class A repeat 7, which is responsible for ligand binding	++
8	Int6	**g.11218193_11218196delGAGT**	**c.940+3_940+6delGAGT**	-	the splicing donor site in exon 6	according to the bioinformatics study may influence the splicing process and possibly lead to the skip of exon 6 and the loss of the extracellular LDLR class A repeat 7	+
9	Ex7	g.11221373G>A	c.986G>A	Cys329Tyr	EGF-like domain 1 (EGF domain)	associated with transport-defective and recycling-deficient receptors; mutations involving cysteine are likely to be associated with domain misfolding	+++
10	Ex7	g.11221441T>C	c.1054T>C	Cys352Arg	EGF-like domain 1	mutations involving cysteine substitutions may be involved in misfolding of this domain, since they may interrupt disulfide bridges that stabilize this kind of domains	++
11	Ex9	g.11224013C>T	c.1246C>T	Arg416Trp	LDLR class B repeat 1	class 5 variant (Etxebarria A, et al., 2015) [5]: mutant	+++

						receptors that fail to release LDL into the sorting endosome	
12	Ex9	g.11224094T>C	c.1327T>C	Trp443Arg	LDLR class B repeat 2	recycling-defective alleles that fail to release the ligands in the endosome and thus do not recycle to the cell surface	++
13	Ex10	**g.11224317T>A**	**c.1465T>A**	**Tyr489Asn**	LDLR class B repeat 3	possibly belongs to class 5 mutations, which are defined as recycling-defective alleles that fail to release the ligands in the endosome and thus do not recycle to the cell surface	+
14	Ex11	g.11226816G>A	c.1633G>A	Gly545Arg	LDLR class B repeat 4; EGF precursor domain (involved in the release of ligands attached to the receptor)	a total retention of LDLR in the endoplasmic reticulum (transport-defective alleles) according to functional study performed for the Gly545Trp variant (Benito-Vicente A, et al., 2015) [6]	++
15	Ex12	**g.11227570A>C**	**c.1741A>C**	**Lys581Gln**	LDLR class B repeat 5	possibly belongs to class 5 mutations, which are defined as recycling-defective alleles and fail to release the ligands in the endosome and thus do not recycle to the cell surface. A diminished release activity (release of bound LDL in response to low pH) of mutated receptors has been demonstrated by functional studies for closely located variant His583Tyr (Beglova N, et al., 2004) [7]. Functional studies performed for created change in the same residue, Lys581Trp, have demonstrated reduced surface expression of LDLR (Zhao Z, et al., 2011) [8].	+
16	Ex12	**g.11227585T>C**	**c.1756T>C**	**Ser586Pro**	LDLR class B repeat 5	this domain is predicted to form a beta-propeller which is critical for LDL release inside the lysosomes (at low pH) and recycling of the receptor	+
17	Ex12	g.11227604G>A	c.1775G>A	Gly592Glu / FH Sicily, FH Foggia-1, FH Naples4	LDLR class B repeat 5	reduction in LDLR activity (Romano M, et al., 2010) [9]	+++
18	Int12	**g.11230765T>G**	**c.1846-3T>G**	-	adjacent to the acceptor splice site of intron 12 of the *LDLR* gene	possible effect on the splicing according to the bioinformatics study	+
19	Int14 - Int16	**g.11232896_11239871del**	**c.2141-966_2390-330del**	**Glu714_Ile796del**	in-frame deletion of *LDLR* exons 15 and 16	a null mutation that fails to produce mature LDLR protein : in-frame deletion of *LDLR* exons 15 and 16, causing the loss of O-linked sugar domain, which has been shown to be crucial for the LDLR protein processing	+++

20	Ex15	g.11233924C>T	c.2215C>T	Gln739*	a region that clustered O-linked oligosaccharides	introduces a premature stop codon at amino acid 739 of LDLR, and leads to formation of a truncated protein lacking 14% of its native mass, though an abnormal transcript can be usually rapidly degraded before translation without protein formation; as a rule, this type of products are non-functional (loss of function)	+++
21	Int16	**g.11238766G>C**	**c.2389+5G>C**	-	an intronic position near the splice donor site of exon 16	affects the splicing process according to the bioinformatics study	++
22	Ex17	g.11240215_11240216insG	c.2416_2417ins G	Val806Glyfs*11	comprises a region with five guanine nucleotides; the presence of this one-nucleotide insertion produces an abnormal transcript that could be rapidly degraded before translation by the nonsense-mediated mRNA decay (NMD) pathway	the loss of many important domains, including the region required for MYLIP-triggered down-regulation of LDLR	+++

c.DNA, coding DNA; Chr DNA, chromosomal DNA; DNA, deoxyribonucleic acid; EGF, epidermal growth factor; Ex, exon; Int, intron; LDL, low density lipoprotein; LDLR, low density lipoprotein receptor; N, mutation number; +++, Pathogenic; ++, Very likely pathogenic; +, Likely pathogenic. The new mutations found in this study are highlighted in bold.

[1] Hobbs HH, Brown MS, Goldstein JL. Molecular genetics of the LDL receptor gene in familial hypercholesterolemia. Hum Mutat. 1992;1(6):445-66.

[2] Thormaehlen AS, Schuberth C, Won HH, Blattmann P, Joggerst-Thomalla B, Theiss S, Asselta R, Duga S, Merlini PA, Ardissino D, Lander ES, Gabriel S, Rader DJ, Peloso GM, Pepperkok R, Kathiresan S, Runz H. Systematic Cell-Based Phenotyping of Missense Alleles Empowers Rare Variant Association Studies: A Case for LDLR and Myocardial Infarction. PLoS Genet. 2015 Feb 3;11(2):e1004855.

[3] Hobbs HH, Leitersdorf E, Leffert CC, Cryer DR, Brown MS, Goldstein JL. Evidence for a dominant gene that suppresses hypercholesterolemia in a family with defective low density lipoprotein receptors. J Clin Invest. 1989 Aug;84(2):656-64.

[4] Russell DW, Brown MS, Goldstein JL. Different combinations of cysteine-rich repeats mediate binding of low density lipoprotein receptor to two different proteins. J Biol Chem. 1989 Dec 25;264(36):21682-8.

[5] Etxebarria A, Benito-Vicente A, Palacios L, Stef M, Cenarro A, Civeira F, Ostolaza H, Martin C. Functional characterization and classification of frequent low-density lipoprotein receptor variants. Hum Mutat. 2015 Jan;36(1):129-41.

[6] Benito-Vicente A, Alves AC, Etxebarria A, Medeiros AM, Martin C, Bourbon M. The importance of an integrated analysis of clinical, molecular, and functional data for the genetic diagnosis of familial hypercholesterolemia. Genet Med. 2015 Dec;17(12):980-8.

[7] Beglova N, Jeon H, Fisher C, Blacklow SC. Cooperation between fixed and low pH-inducible interfaces controls lipoprotein release by the LDL receptor. Mol Cell. 2004 Oct 22;16(2):281-92.

[8] Zhao Z, Michaely P. Role of an intramolecular contact on lipoprotein uptake by the LDL receptor.Biochim Biophys Acta. 2011;1811(6):397-408. doi: 10.1016/j.bbalip.2011.04.002.

[9] Romano M, Di Taranto MD, D'Agostino MN, Marotta G, Gentile M, Abate G, et al.. Identification and functional characterization of LDLR mutations in familial hypercholesterolemia patients from Southern Italy. Atherosclerosis. 2010;210(2):493-6.

Table S4. Mutation effect prediction algorithms – missense mutations in *LDLR*.

N	*LDLR* variant	Mutation effect prediction algorithms			
		SIFT	Polyphen-2 (HumVar)	Polyphen-2 (HumDiv)	MutationTaster
5	Pro181Leu	Tolerated	Possibly damaging	Possibly damaging	Disease-causing
4, 7, 9-17	Ser177Leu, Glu308Lys, Cys329Tyr, Cys352Arg, Arg416Trp, Trp443Arg, Tyr489Asn, Gly545Arg, Lys581Gln, Ser586Pro, Gly592Glu	Damaging	Probably damaging	Probably damaging	

LDLR, low density lipoprotein receptor; N, mutation number.

Table S5. Mutation effect prediction algorithms – splice-site mutations in *LDLR*.

N	*LDLR* variant	Mutation effect prediction algorithms
8	c.940+3_940+6delGAGT	Five software tools (SSF, HSF, MaxEnt, GeneSplicer, and NNSplice) showed that HSF predicted a significant (> 20%) change in its score, other software tools predicted the loss of the native donor site in exon 6.
18	c.1846-3T>G	Five software tools (SSF, HSF, GeneSplicer, MaxEnt and NNSplice) suggest a possible effect on the splicing in the acceptor splice site of intron 12. NNSplice and GeneSplicer predicted the disappearance of the acceptor site. SSF, MaxEnt and GeneSplicer showed significant decreases of the scores.
21	c.2389+5G>C	Five software tools (SSF, HSF, GeneSplicer, MaxEnt, and NNSplice) suggest a possible effect on splicing in an intronic position near the splice donor site of exon 16. SSF predicts the disappearance of the donor site. The rest of predictors, except for HSF, show significant decreases of the scores.

LDLR, low density lipoprotein receptor; N, mutation number.

Table S6. Cardiovascular risk factors and presence of myocardial infarction in patients with heterozygous familial hypercholesterolemia.

		Risk factors	With MI<br>(n = 17)	Without MI<br>(n = 31)	p
Risk factors	Unmodified	Presence of pathogenic mutation	8 (38%)	13 (62%)	NS
		Age	47.0 (44.0-63.0)	53.0 (43.0-61.0)	NS
		Gender (male/female)	12/5 (25%/10%)	13/18 (27%/38%)	0.08
	Modified	Arterial hypertension	12 (41%)	17 (59%)	NS
		Smoke history<br>No/Yes/Gave up	6/6/5	16/6/9	NS
		Obesity	6 (40%)	9 (60%)	NS
		Max TC, mmol/L<br>Max LDL-C, mmol/L	9.4 (9.0-12.0)<br>7.5 (6.7-8.9)	10.6 (9.6-12.0)<br>8.6 (7.6-10.0)	NS<br>NS
		Current TC, mmol/L<br>Current LDL-C, mmol/L	9.4 (8.0-10.1)<br>7.0 (5.9-8.2)	10.1 (9.2-11.9)<br>8.3 (6.7-9.4)	0.08<br>0.06

MI, myocardial infarction; TC, total cholesterol. NS, $p \geq 0.1$.
